# Social capital and changes of psychologic distress during early stage of COVID-19 in New orleans

**DOI:** 10.1038/s41598-024-56249-1

**Published:** 2024-03-09

**Authors:** Kimberly Wu, Erica Doe, Gabriella D. Roude, Jasmine Wallace, Samantha Francois, Lisa Richardson, Katherine P. Theall

**Affiliations:** 1 School of Public Health and Tropical Medicine, Tulane University, New Orleans, USA; 2 Institute of Women and Ethnic Studies, New Orleans, USA; 3 Clark University, Worcester, USA

**Keywords:** Risk factors, Human behaviour

## Abstract

Here we report on the relationship between measures of social capital, and their association with changes in self-reported measures of psychological distress during the early period of the COVID-19 pandemic. We analyze data from an existing cluster randomized control trial (the *Healthy Neighborhoods Project*) with 244 participants from New Orleans, Louisiana. Changes in self-reported scores between baseline (January 2019–March 2020) and participant’s second survey (March 20, 2020, and onwards) are calculated. Logistic regression is employed to examine the association between social capital indicators and measures of psychological distress adjusting for key covariates and controlling for residential clustering effects. Participants reporting higher than average scores for social capital indicators are significantly less likely to report increases in psychosocial distress between pre and during the early stage of the COVID-19 pandemic. Those who report higher than average sense of community were approximately 1.2 times less likely than those who report lower than average sense of community scores to experience increases in psychological distress before and during the global pandemic (OR 0.79; 95% CI 0.70,0.88, p ≤ 0.001), even after controlling for key covariates. Findings highlight the potentially important role that community social capital and related factors may play in the health of underrepresented populations during times of major stress. Specifically, the results suggest an important role of cognitive social capital and perceptions of community membership, belonging, and influence in buffering changes of mental health distress experienced during the initial period of the COVID-19 pandemic among a sample of residents.

## Introduction

According to the National Institute of Mental Health (NIMH) data from 2021, over 57 million individuals in the United States live with mental illness^1^ A Kaiser Family Foundation analysis of U.S Census Data found that the percentage of adults reporting anxiety and depressive symptoms increased during the first year of the COVID-19 pandemic from 35.9% in April 2020 to 39.3% in February 2021^2^. While it is difficult to measure the full impact of the pandemic on the mental health of Americans, recent studies have pointed to moderate increases of clinical anxiety and depression in the first year of the pandemic as compared to prevalence data from 2017 to 2019^3^. However, the same research team also raise the possibility that the findings based on aggregate data may be masking trends for more specific subgroups such as first responders^3^.

More nuance is needed, especially when considering the mental health implications of historical and structural inequities in the United States. Evidence of higher mental health stress, anxiety, depression, and worry among Black, Indigenous, and other people of color (BIPOC) have been documented during the pandemic^4–6^. Such disparities can be attributed to a range of structural determinants that impact one’s ability to navigate disasters. This includes stress from COVID related financial instability as well as grief experienced as a result of a loss of loved one unexpectedly during the pandemic^7^. Furthermore, people of color were more exposed to risk of infection and death, in part due to higher rates of chronic conditions among BIPOC individuals, and also in part due to their roles as essential workers, with Black individuals most represented in high-risk positions^7,8^.

Research of the racial and ethnic disparities in mental health and mental healthcare during the pandemic from April 2020 April 2021, raises the finding that Black, Hispanic, and Asian adults experienced worsening mental health before and during the pandemic, especially when considering the impacts of the murder of George Floyd and the six Asian women in Atlanta^9^. The authors attribute such mental health disparities to limited access to mental health professionals and removal of sources for coping that include informal supports, religious organizations, and contact with extended family and kin during the pandemic^9,10^. Therefore, additional research to understand and bolster local mechanisms for fostering social connections and action when navigating emergencies and their recoveries are avenues that requires continued attention^9,10^.

Research to better understand mechanisms that may buffer or mitigate adverse mental health symptoms during disasters such as the COVID-19 pandemic highlight the importance of social supports and social connections^11,12^. Mannarini and team’s study found that sense of community mitigated the stress experienced during the pandemic and was positively associated to well-being^12^. Aspects of social relationships were drastically affected by changes during the early stages of the pandemic, including major shifts to patterns of socialization and employment as a result of stay-at-home orders, school shutdowns, and upholding guidance related to physical distancing. While there is evidence of engaging alternative spaces for socializing, including the unique role of social media and technology, there are still barriers faced by communities in neighborhoods with limited access to public spaces, poor conditions of public spaces, or concerns of crime and safety^13–15^.

Given the impact of the challenges presented by the pandemic on mental health and the important role that neighborhood relationships may play in buffering or exacerbating pandemic related stress, this study aims to explore how dimensions of social capital (operationalized as sense of community and neighborhood collective efficacy) are associated with changes in self-reported psychological distress and perceived stress among a sample of primarily Black residents living in low-income neighborhoods in New Orleans, Louisiana–an area that experienced great inequities in rates of COVID-19 infection, morbidity, and mortality^16^. On the tail of Mardi Gras in February 2020, New Orleans was one of the early epicenters of the pandemic, making national headlines and reporting significantly higher death rates than Seattle and New York City at the time^17^.

This research focuses on the impact of two dimensions of social capital- sense of community (SOC) and collective efficacy. Building on the work of Perkins and Long^18^ and their four components of social capital, sense of community and collective efficacy are presented as the “cognitive or intrapsychic components of social capital”, with SOC reflected in informal contexts and collective efficacy in formally organized contexts. Sense of community, as defined by McMillan and Chavis is operationalized as a composite of four dimensions: (1) fulfilling needs that can be met by a community, (2) group membership and sense of belonging to a community, (3) influence and ability to make an impact on a community, and (4) emotional connection or feeling of bonding^19^. Empirical findings raise the mental health benefits of sense of community, both to promote and buffer from adverse effects^20–22^. Collective efficacy is defined as “trust in the effectiveness of organized community action” by Perkins and Long and has been found to be negatively associated with depression and depressive symptoms, especially among older adults^18,23–25^. This conceptualization of indicators of social capital as sense of community and collective efficacy highlights the importance of studying social relationships and their connections to health outcomes. Furthermore, focusing on indicators of social capital within neighborhood spaces at a collective level presents opportunities for understanding how relationships among neighbors and within local networks can have an effect on mental health and psychological distress.

In this study, we examine how community variables of SOC and CE among residents living in areas of New Orleans with some of the highest rates of violence and poverty affect changes in self-reported psychological distress and perceived stress following the first several months into the COVID-19 pandemic. Our hypothesis was that residents with higher levels of social capital, operationalized as measures of perceived sense of community and collective efficacy, experience lower levels of changes to self-reported distress and perceived stress in the periods right before and during the early stages of the COVID-19 pandemic.

## Results

Slightly more than half of the total sample population reported experiencing an increase in psychological distress between baseline and Wave 2 data collection. Table 1 presents the sociodemographic background data of individuals involved in the study, stratified by changes in psychological distress from the pre-COVID onset and during the pandemic. The average age of participants was 52 years. When stratifying by 10-year age categories, we can see that at least half of respondents in the age groups of 20–29 (53.9%), 40–49 (50%), and 60–69 (54.8%) experienced an increase in psychological distress. The only age group with a majority of respondents experiencing no change or decrease in psychological distress were among those 70 and above (75%). About 70% of the sample identified as female and 30% as male.

**Table 1:** Individual and neighborhood socio-demographic characteristics of study participants stratified by psychological distress before and during COVID 19^a^.

	Increase in psychological distress (N = 116)	No change or decrease in psychological distress (N = 128)	Total (N = 244)
	Mean (range)/(%)	Mean (range)/(%)	Mean (range)/(%)
Average age (years)	50.90 (22–83)	53.38 (22–94)	52.20 (22–94)
Age (10 year period groups)			
20–29	53.85%	46.15%	10.66%
30–39	46.34%	53.66%	16.80%
40–49	50%	50%	16.39%
50–59	46.81%	53.19%%	19.26%
60–69	54.84%	45.16%	25.41%
70 and above	25%	75%	11.48%
Sex*			
Female	69.83%	69.53%	69.67%
Male	30.47%	30.17%	30.33%
Self-reported ethnicity			
Non-Hispanic Black	77.59%	77.34%	77.46%
Non-Hispanic white	12.93%	16.41%	14.75%
Non-Hispanic Other	5.17%	3.13%	4.10%
Other (Hispanic)	4.31%	3.13%	3.69%
Relationship status			
Married/living with a partner	26.72%	24.22%	25.41%
Divorced/Separated	9.48%	12.50%	11.07%
Widowed	10.34%	10.16%	10.25%
Single	38.79%	39.84%	39.34%
Other (Multiple/Never married)	14.66%	13.93%	13.93%
Employment status			
Full-time	37.93%	39.06%	38.52%
Part-time	12.93%	16.41%	14.75%
Unemployed	9.48%	4.69%	6.97%
Unable to work due to disability	14.66%	17.19%	15.98%
Other	25.00%	22.66%	23.77%
Education			
Less than high school	5.31%	13.71%	9.70%
High school graduate	32.74%	26.61%	29.54%
Some college	36.28%	24.19%	29.96%
4 year college	17.70%	22.58%	20.25%
Graduate or professional school	7.96%	12.90%	10.55%
Average perceived neighborhood collective efficacy*^b^	26.10	28.94	27.57
Average sense of community index score^c^	8.96	10.36	9.69

Mean/(%) of any variable based on < 10% missing.

*Statistically significant difference at p < 0.05.

^a^Psychological distress measured by Kessler Psychological Distress Scale with scores ranging from 10–50 where higher scores indicate greater distress.

^b^Percevied neighborhood collective efficacy eight items measured on a five-point scale where higher scores indicate higher collective efficacy.

^c^Sense of community index measures the aggregate score of true/false (true = 1, false = 0) responses for 12 items that capture subscale responses of sense of membership, integration, influence, and shared emotional connection with scores ranging from 0–12.

A majority of the sample self-identified as non-Hispanic Black/African American (77%), roughly 15% identified as non-Hispanic white, with 4% identifying as non-Hispanic other, and the remaining 4% identifying as other (ethnically Hispanic). Nearly 40% of study participants identified as single, a little over 25% identified as married or living with a partner, 10% identified as widowed, 11% identified as divorced or separated, and the remaining 14% identified being in other types of relationship statuses. Almost 39% of the participants identified having full-time employment. Among respondents who reported an increase in psychological distress, 10% reported experiencing unemployment, as compared to 5% of respondents who reported a decrease or no change in psychological distress before and during the pandemic.

From t-test analysis, the average perceived neighborhood collective efficacy score was 28 (range from 8 to 40), with scores slightly lower for participants who experienced an increase in distress from before-during the pandemic (p ≤ 0.05). Furthermore, the average sense of community score was a little over 9 (range from 0 to 12), with those who reported experiencing an increase in distress scoring slightly lower scores (although the association was not significant).

Table 2 presents the results from crude and adjusted logistic regression models for each marker of social capital (sense of community and collective efficacy). From the crude models, respondents reporting higher levels of sense of community were significantly less likely to experience an increase in psychosocial outcomes over time for both measures of psychological distress (K6) (OR 0.51, 95% CI 0.29, 0.91, p ≤ 0.05) and perceived stress (PSS) (OR 0.49, 95% CI 0.28, 0.87, p ≤ 0.05). Crudely, respondents who reported higher levels of neighborhood collective efficacy also reported lower odds of increased psychological distress over time for both K6 (OR 0.40, 95% CI 0.23, 0.71, p ≤ 0.05) and PSS (OR 0.69, 95% CI 0.40, 1.19) measures.

**Table 2:** Impact of social capital on psychologic distress and perceived stress differences before and during COVID-19: results of crude and adjusted logistic models.

	Crude models		Adjusted models^a^	
	Odds ratio	95%CI	Odds ratio	95% CI
Psychological distress differences outcome (K6)				
Model 1: Sense of community	0.51*	(0.2911, 0.9054)	0.79*§*	(0.7038, 0.8834)
Model 2: Neighborhood collective efficacy	0.40*	(0.2292, 0.7099)	0.90*§*	(0.8523, 0.9543)
Perceived Stress differences outcome (PSS)				
Model 3: Sense of community	0.49*	(0.2824, 0.8652)	0.84**	(0.7458, 0.9488)
Model 4: Neighborhood collective efficacy	0.69	(0.4011, 1.1867)	0.92**	(0.8739, 0.9747)

Modeling difference score for both K6 and perceived stress difference. A greater difference score indicates an increase in psychological distress and perceived stress following the start of the COVID pandemic.

*statistical significance at p < 0.05.

**statistical significance at p ≤ 0.01.

^§^statistical significance at p ≤ 0.001.

^a^Controlling for age categories, sex, relationship status, education, employment, attitudes around COVID-19 prevention, and cluster-level effects.

When adjusting for the effects of age, sex, relationship status, education level, employment, attitude around COVID-19 prevention, and cluster-level design, both measures of social capital continued to exhibit significant and protective associations with changes in psychosocial outcomes. Study participants who reported higher-than-average sense of community scores were approximately 1.2 times less likely than those who reported lower-than-average sense of community scores to experience increases in psychological distress before and during the global pandemic (OR 0.79; 95% CI 0.70,0.88, p ≤ 0.001) when controlling for covariates. Furthermore, the same participants were also slightly less likely than those who reported lower-than-average sense of community scores to experience increased perceived stress differences (OR 0.84; 95% CI 0.75, 0.95, p ≤ 0.01).

From the models, higher neighborhood collective efficacy scores were associated with significantly less increases in psychosocial outcomes between prior and during the early stages of the COVID-19 pandemic, although the effects were smaller. Participants who reported higher-than-average neighborhood collective efficacy scores were 10% less likely than those who reported lower-than-average scores to experience increases in distress over time (OR 0.90, 95% CI 0.85, 0.95, p ≤ 0.001) when controlling for relevant variables. In addition, participants with higher-than-average neighborhood collective efficacy scores were 8% less likely than those who reported lower-than-average-scores to experience increased perceived stress scores over time (OR 0.92, 95% CI 0.87, 0.97, p ≤ 0.01).

## Discussion

This study contributes to the growing literature on the role that social capital may play in buffering pandemic stress or in other emergency contexts, with both indicators of social capital reflecting modest protective effects from increased psychological distress and perceived stress before and during the early stages of the COVID-19 pandemic. Our models showed that residents who indicated higher measures of sense of community and collective efficacy were also less likely to report increases in psychological distress and perceived stress during the transition into the earlier periods of the global pandemic. Although the general consensus on the relationship between social capital and its effects on mental health is mixed, these results corroborate findings supporting the positive role of social capital on mental health in community contexts^26^ and do so in a sample of adults living in some of the most violent and deprived areas of a southern city. Related studies have reported findings where lower levels of community social capital were associated with higher psychological distress.^27,28^. The benefits of social relationships at a collective level may be connected with facilitating positive physiological and cognitive responses that minimize stress or partaking in risky behaviors^29^.

Our findings also add to the literature that supports the role of social capital on outcomes related to the COVID-19 pandemic and experiences during crisis situations^30^. Psychological responses to navigating “home confinement” and quarantine during the early stages of the pandemic have been documented, and includes negative emotional outcomes and stress when managing the burdens and logistics of securing day-to-day supplies, finances, and other familial needs^31^. In response, fostering social capital through strengthening community and familial connections is recognized to promote more trust and sharing of information to inspire preventative action and preparation beneficial to navigating changes during this time^30,32^. In addition, social capital is theoretically linked to promoting civic norms outside of formal institutions, and may serve to unite residents to work towards shared goals and fostering contributions towards collective action when navigating shared stressors^32^.

Finally, this study adds to the existing literature by including experiences of racial and ethnic minoritized groups during the early periods of the pandemic^33^. From a study in the UK, researchers identified that social capital and psychological distress were distributed differently across Black and ethnic minorities, raising the importance of considering diverse experiences across gender and racial identities^33^. While this study does not analyze outcomes by racial and ethnic subgroups, it focuses on a sample of predominantly Black female residents living in New Orleans, Louisiana. Specific to New Orleans, COVID-related mandates, and phases of the city’s reopening since March 2020 have had varying impacts on the economic, social, and health equity-related outcomes^34^. Black men and women representing a majority of the city’s population were disproportionately represented among the ranks of essential and frontline workers in New Orleans, and Black women have been further affected by job losses related to COVID-19 and lower wages for their labor^35^. Additional research into the nuances of social networks and specific mechanisms of buffering for psychological distress are relevant to better understand the health realities and priorities for Black residents in New Orleans, Louisiana, and the Gulf South at large^36^.

Our findings reflect a protective element of social capital among respondents who reported higher than average sense of community and collective efficacy scores. An area of critique related to social capital raises the need for greater nuance to tease out underlying mechanisms that motivate the formation of social connections and networks^37^. This study was not able to address this critique directly, but our findings provides us with directions for future projects through the incorporation of ethnic identity measures and validated scales to assess the potential moderating effects of perceived racial or ethnic identities and relationships to social capital and mental health.

One mechanism that may bridge individual social capital to community social capital within the Black community in New Orleans may be connected to strong historical and contemporary practices of grassroots mutual aid efforts, especially in the face of managing disasters such as in the cases of Hurricane Katrina (2005) and recently Hurricane Ida (2021)^38,39^. Mutual aid is a form of civic participation, where people take on the responsibilities for direct care for one another beyond symbolic acts and into creating new relational patterns and structures to meet immediate needs^40^. Mutual aid has garnered more national attention during COVID-19 through the work of local institutions and groups led by communities of color, many of whom are directly affected by structural inequities, and have continued to sustain their efforts to provide resources to community members^40,41^. Raising visibility of the organized capacity of marginalized groups to exercise agency and materially provide for each other’s needs is an important narrative to counter claims that groups of color are “dependent” on institutions and labeled “resource poor” in a way that perpetuates unbalanced stereotypes and perceptions of communities of color^38^.

There are also several limitations to this study that must be raised. Primarily, the findings were drawn from a small and select sample of residents and are based on self-reported data which is subject to recall and social desirability biases. Findings from the study cannot be generalizable to all residents in New Orleans but serve as a helpful reminder to invest in more holistic conceptions of mental health that draw connections between individuals and community life. While the sample size was small, it is representative of many low-income neighborhoods in the city. There are also limitations to our indicators of social capital, as they do not capture the full conceptualization of the construct. An ongoing consideration related to operationalizing social capital includes clarifying the type and level of focus of the measures utilized. Generally, measures of sense of community and collective efficacy fall within cognitive forms of social capital that capture community-level characteristics as opposed to individual-level traits^42^. As demonstrated from our findings, the sense of community index was found to be a stronger buffer of psychosocial change scores as compared to the measure for neighborhood collective efficacy among the sample population. Neighborhood collective efficacy may differ from sense of community by capturing dimensions of neighborhood capacity that extend beyond perceptions of social connection into the realm of engagement in shared action. Finally, there is a lack of COVID-19 related measures in the first two waves of the survey, which would be important to consider for future analysis.

Findings from this study highlight the value of conducting additional research into the relationship of measures of social capital and particular factors most relevant to underrepresented populations to gain a better understanding of the pandemic’s impact and influence. Specifically, the results suggest an important role of cognitive social capital and perceptions of community membership, belonging, and influence in buffering changes of mental health distress experienced during the initial period of the COVID-19 pandemic among a study sample that is majority Black and female across 23 neighborhoods of New Orleans, Louisiana. Furthermore, incorporating social capital and its relevance for highlighting existing and long-standing grassroots and community structures are useful for identifying norms and practices that address local needs and provide forms of mental health coping^37^.

## Methods

The study draws on secondary survey data created for an existing cluster randomized control trial funded by the National Institute of Health (NIH, R01HD095609) with additional funding support from the Robert Wood Johnson Foundation’s Evidence for Action Program. The parent study, referred to as the *Healthy Neighborhoods Project (HNP),* includes 23 neighborhoods in New Orleans, and is aimed at testing the impact of neighborhood beautification on violence prevention and overall health. The parent study includes a longitudinal cohort portion that follows the experiences of neighborhood residents throughout the duration of the 5-year project. Publications of survey data from the parent study are currently in development. Participants for the survey were recruited from the 194 randomly selected clusters (101 intervention clusters and 93 control clusters) using a 1/8-mile radius and in areas of the city with the highest levels of violent crime and poverty rates more than 30%. All residents from these clusters were invited to participate through the use of mailers sent to valid addresses. The current study sample is a sub-sample of 244 respondents with baseline data collected before the COVID-19 pandemic (January 2019-early March 2020), and Wave 2 data collected from March 20, 2020 and onwards^43^. The survey was pilot tested with a sample of 5 residents prior to its launch. All methods in the study are in accordance with relevant guidelines and regulations.

### Data collection

Trained interviewers collected survey data utilizing RedCap^™^ software in person (5%, n = 12) and over the phone (95%, n = 232). There are no differences in sociodemographic background, exposure, or outcomes of interest between participants who completed in one modality comparted to the other. Baseline and Wave 2 surveys took approximately 45 min to complete, and informed consent was obtained from all the participants. Participants were removed from the study sample if they engaged in forms of careless responding. This is defined as (1) spending less than 5 seconds per item (calculated based on survey completion time and the total number of items) and (2) responding to all items with maximum values (lacking variance). This study received approval from Tulane University’s Institutional Review Board.

### Measures

#### Dependent variables

To capture differences in self-reported mental health distress and stress, the Kessler 6 (K6) Psychological Distress Scale and Perceived Stress Scales (PSS) were included in the parent study. The K6 scale measures the frequency of “non-specific psychological distress” using six items on a Likert scale, with each item ranging from zero for “none of the time” to four for “all of the time”^44^. Questions focus on negative feelings/emotions and the related ability to carry out normal activities and care-seeking^44^. The items are reduced into a summary score, with higher scores indicating greater psychological distress. Scores of 5 and over indicate moderate psychological distress and 13 and over indicate serious psychological distress^44^. The index demonstrated good internal reliability in the parent sample (Cronbach’s alpha = 0.89).

The PSS was measured using four items on a Likert scale, where each item was scored ranging from zero for “never” to four for “very often”. Positive items were reverse coded and then a total score was obtained by aggregating across all 4 items^45^. The scale also demonstrated acceptable internal reliability in the parent sample (Cronbach’s alpha = 0.71). To examine change in self-reported mental health, difference scores for K6 and PSS scores were calculated between each participant's baseline (pre-pandemic) and Wave 2 (during pandemic) responses. An increase in change scores for either measure indicated an increase in self-reported mental health distress or perceived stress over time and was coded as one to indicate an increase or zero to indicate no change/decrease.

#### Independent variables

Social capital was assessed with two separate indicators—*Sense of Community* and *Collective Efficacy*. Sense of community was measured using the sense of community index (SCI), a validated tool that measures perceptions of connection and membership to a group or community. The SCI includes 12 items, scored on a “mostly true/mostly false” scale for each item, resulting in total scores ranging from zero to twelve. Lower scores indicate a lower perception of a sense of community^46^. The SCI also included two subscales to measure emotional connection to community as well as sense of belonging to community or membership. Each subscale was made up of six questions^46^. The SCI scale was designed to include an overall score and subscales scores that capture more specific constructs for sense of membership, sense of influence, reinforcement of needs, or shared emotional connection^46^. Scale items included responding to statements such as, “I can recognize most of the people who live in my neighborhood”, “I care about what my neighbors think of my actions”, “I have influence over what this neighborhood is like”, and “If there is a problem in this neighborhood people who live here can get it solved”. Within our sample, the SCI demonstrated strong reliability (Cronbach’s alpha = 0.85).

In addition to incorporating the SCI, neighborhood collective efficacy was assessed with survey items taken from measures developed by Sampson and colleagues^47^ which included 8 items scored on a 5-point scale ranging from 1 (“strongly disagree”) to 5 (“strongly agree”), for total scores ranging from 8 to 40. To measure collective efficacy, participants responded to statements such as, “You can count on adults in this neighborhood to watch out that children are safe and don't get into trouble”, “The police protection in my neighborhood is adequate”, “I can trust the government in New Orleans to do what is right for my neighborhood”, and “If there is a problem around here, the neighbors get together to deal with it”. Lower scores indicated a lower sense of perceived neighborhood collective efficacy. The measure demonstrated strong reliability in our sample (Cronbach’s alpha = 0.85).

#### Covariates

The covariates included in the study are related to the sociodemographic backgrounds of the participants. These variables included age, sex, relationship status, education, employment status, and participant attitudes about COVID-19 prevention. *Age* was measured continuously and ranged from 20 to 80 years. The responses were recoded into 9-year categories, “20–29”, “30–39”, “40–49”, “50–59”, “60–69”, and “over 70” to organize into ordinal categories. *Sex* was measured as a binary variable where 1 indicates “female”, and 0 indicates “male”. *Relationship status* was measured in the categories of “married”, “living with a partner”, “divorce/separated”, “widowed”, “single/never married”, and “multiple”. A response of “multiple” indicates that the respondent may experience multiple relationship statuses at once. The categories of “living with a partner” were combined with “married” and, “never married” with “multiple” and “other”, to address the small percentage of responses (less than 5%). Education attainment included categories of “less than high school education”, “high school/GED”, “some college”, “college”, and “graduate” levels. *Employment status* was recoded so that each category had more than 5% of the sample size for analysis. The categories are “full time”, “part-time”, “unemployed”, “unable to work because of a disability”, and “other”. Other included individuals who identified as retired, full-time homemakers, and individuals in school or a training program. *Attitude around COVID-19 prevention* was measured as participant’s response to the statement “If I don't take preventative action, then I am likely to get COVID-19.” Participants responses were recoded into three categories- “probably true”, “probably false”, and “not sure/no opinion”.

#### Data Analysis

Descriptive, bivariate, and multivariate analyses were conducted, including chi-square and t-test for categorical and continuous bivariate analyses, respectively. Checks for multicollinearity were performed for independent variables. Logistic regression was employed to examine the association between social capital indicators and measures of psychological distress (K6 and PSS), adjusting for key covariates. Regression models were analyzed separately for each measure of social capital and mental health outcome—increases in psychological distress or perceived stress—before March 2020 and during the COVID-19 pandemic from March 2020 vs. no change or decrease. All analyses were performed with Stata/BE 17.0.

## Data Availability

The repository for the related dataset can be found at the following Box link: https://tulane.box.com/s/ox7h2i4isp8ri7euula6b969blux6585.
